# γ-TEMPy: Simultaneous Fitting of Components in 3D-EM Maps of Their Assembly Using a Genetic Algorithm

**DOI:** 10.1016/j.str.2015.10.013

**Published:** 2015-12-01

**Authors:** Arun Prasad Pandurangan, Daven Vasishtan, Frank Alber, Maya Topf

**Affiliations:** 1 Institute of Structural and Molecular Biology, Birkbeck College, University of London, Malet Street, London WC1E 7HX, UK; 2 Division of Structural Biology, Oxford Particle Imaging Centre, Wellcome Trust Centre for Human Genetics, University of Oxford, Oxford OX3 7BN, UK; 3 Program in Molecular and Computational Biology, University of Southern California, 1050 Childs Way, RRI413E, Los Angeles, CA 90089, USA

## Abstract

We have developed a genetic algorithm for building macromolecular complexes using only a 3D-electron microscopy density map and the atomic structures of the relevant components. For efficient sampling the method uses map feature points calculated by vector quantization. The fitness function combines a mutual information score that quantifies the goodness of fit with a penalty score that helps to avoid clashes between components. Testing the method on ten assemblies (containing 3–8 protein components) and simulated density maps at 10, 15, and 20 Å resolution resulted in identification of the correct topology in 90%, 70%, and 60% of the cases, respectively. We further tested it on four assemblies with experimental maps at 7.2–23.5 Å resolution, showing the ability of the method to identify the correct topology in all cases. We have also demonstrated the importance of the map feature-point quality on assembly fitting in the lack of additional experimental information.

## Highlights

• γ-TEMPy uses a genetic algorithm to fit multiple components into 3D-EM density maps
• The fitness score is a combination of a Mutual Information score and a clash penalty
• Efficient sampling is aided by using map feature points from vector quantization
• Native topologies for assemblies containing up to eight components can be predicted

## Graphical Abstract

**Figure:**
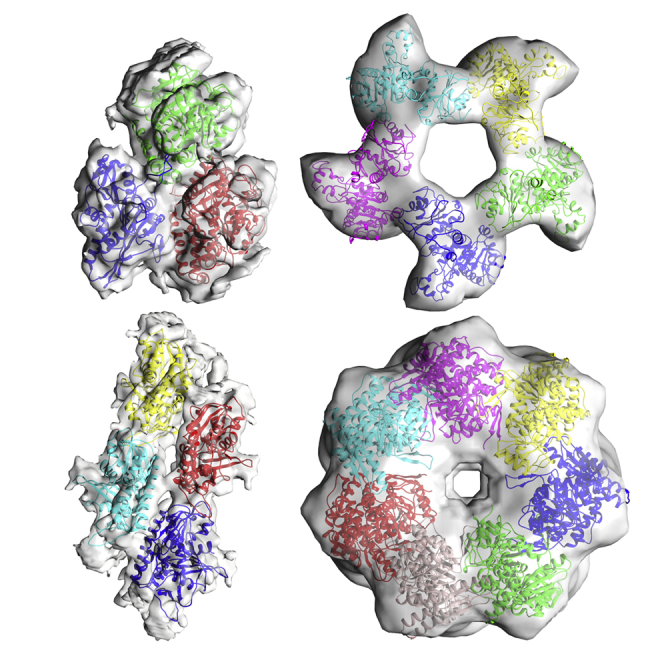

## Teaser

Pandurangan et al. developed a genetic algorithm to simultaneously fit multiple atomic components into low-resolution 3D-EM density maps. The method was tested on simulated and experimental benchmarks with resolutions between 10 and 23.5 Å. It identifies native topologies for assemblies containing up to eight components.

## Introduction

Protein and nucleic acid assemblies are central to the workings of the cell, and a great deal of understanding is gained from determining the structures, interfaces, and interactions of their components. X-Ray crystallography has been the mainstay of such studies, but cryoelectron microscopy (cryo-EM) is increasingly being used to characterize large and heterogeneous complexes that are difficult to study by other techniques (Cheng, 2015, Elmlund and Elmlund, 2015, Lander et al., 2012, Thalassinos et al., 2013). In particular, cryoelectron tomography combined with subtomogram averaging allow for the structure determination of macromolecular machinery in near-native contexts (for instance when they are membrane-bound), which is difficult to achieve with other methods (Zeev-Ben-Mordehai et al., 2014). However, the low resolutions characteristic of such reconstructions make interpretation of atomic interfaces impossible without integrating information from other higher-resolution studies.

Many computational methods have been developed to help fit atomic models of individual components from crystallography, nuclear magnetic resonance, or structure prediction into low-resolution density maps (Esquivel-Rodriguez and Kihara, 2013, Thalassinos et al., 2013, Villa and Lasker, 2014). Such methods can be broadly classified into flexible fitting (Topf et al., 2008), whereby the conformation of the atomic model is considered partially malleable, and rigid fitting (Roseman, 2000), whereby the conformation of each model remains fixed. Most of these methods are designed to optimize the fit of a single component into a density map, even if the map is of a larger assembly. Ideally, the available techniques can be extended to address the problem of fitting multiple components simultaneously into the assembly density maps (assembly fitting). The immediate difficulties in such implementations include the huge increase in the configuration search space and the need to score multi-component interactions in addition to the similarity between the atomic model and EM map. The number of configurations available to find an optimal fit for three-component assembly (with a given search radius of 360° with step size of 10°) is of the order of 10^14^. This is only considering rotational moves, given the initial placement of components. If we consider the translational position of each component, the number of configurations to be explored would be far larger. Therefore, one needs to use heuristic methods to intelligently reduce the configuration space and search it efficiently. Thus, to efficiently identify the optimal solution, assembly fitting requires an efficient global optimization technique coupled with a robust scoring scheme.

A few tools have been developed for assembly fitting. These include techniques based on exhaustive sampling (Birmanns et al., 2011, Kawabata, 2008), combinatorial optimization using a divide-and-conquer approach (Lasker et al., 2009), multiple protein docking procedure using the 3D Zernike descriptor (Esquivel-Rodriguez and Kihara, 2012), and point set matching using integer quadratic programming (Zhang et al., 2010). Most methods use a density-based cross-correlation score to measure the goodness of fit, in combination with scores borrowed from protein-protein docking to favor inter-component interactions and penalize non-favorable interactions (Lasker et al., 2009). In some methods, symmetry restraints are applied where appropriate (Kawabata, 2008, Lasker et al., 2009). In the absence of symmetry, assembly fitting becomes even more challenging. It has been shown that the use of additional experimental constraints can improve the predictions (van Zundert et al., 2015).

Since the configuration space is so immense, an exhaustive sampling is not feasible. Heuristic methods that aim to find optimal or good solutions by examining only a fraction of the possible candidate solutions serve as a good alternative to the exhaustive sampling approach for finding the global optimum. One particular global optimization technique of interest is the genetic algorithm (GA), a heuristic search method that seeks to emulate the process of natural selection (Goldberg, 1989). GAs have been applied to various problems in structural biology, for example in ab initio modeling (Arunachalam et al., 2006, Contreras-Moreira et al., 2003), protein-protein docking (Gardiner et al., 2003), comparative protein structure modeling (John and Sali, 2003), fitting models into small-angle X-ray scattering profiles (Chacon et al., 2000), and, more recently, in EM density fitting (Esquivel-Rodriguez and Kihara, 2012). Here, we apply a GA for the purposes of assembly fitting called γ-TEMPy (Genetic Algorithm for Modeling Macromolecular Assemblies with Template and EM comparison using Python). γ-TEMPy is developed from the TEMPy Python package (Farabella et al., 2015). Most of the assembly-fitting methods described above use the cross-correlation coefficient to measure the goodness-of-fit. Here, for the first time, we use a mutual information score (Vasishtan and Topf, 2011) within such​ context.

We begin by describing the details behind the γ-TEMPy algorithm. We then demonstrate its performance on a benchmark of simulated and experimental cases. Finally, we discuss the implications of γ-TEMPy for the structural characterization of large assemblies.

## Results

### Theory

**Figure 1:**
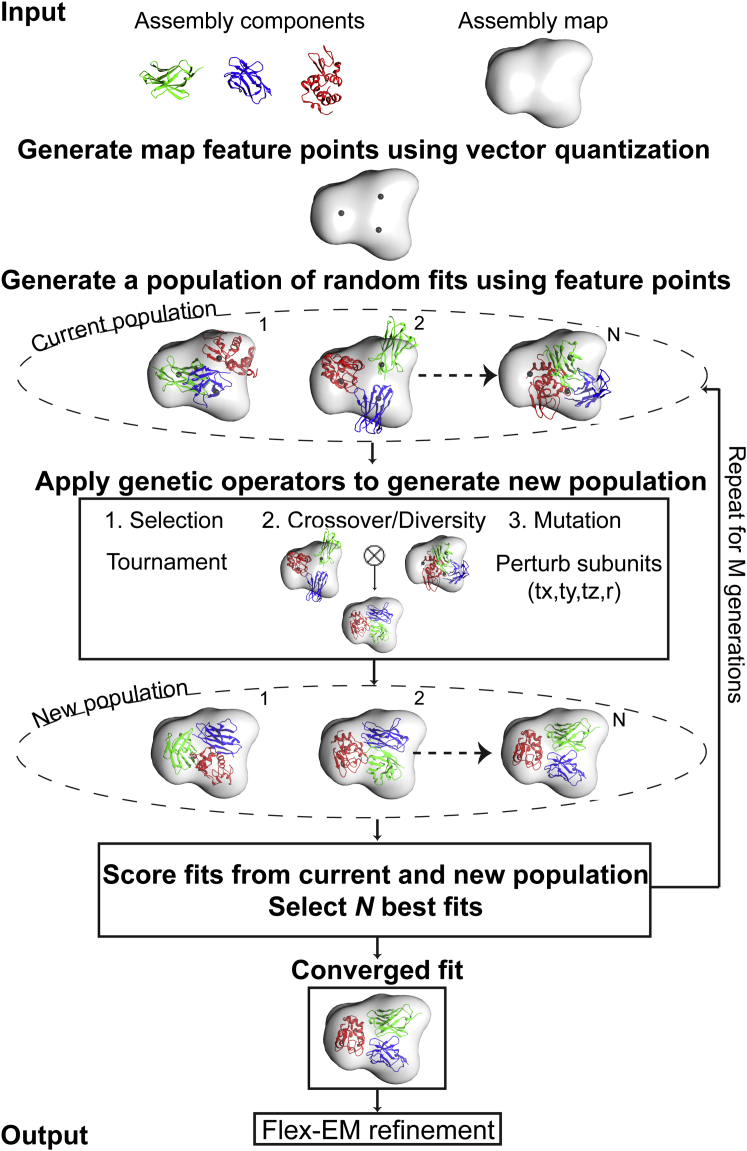
Schematic Diagram Describing the Process of Fitting Multiple Components into the Assembly Maps Using a Genetic Algorithm The method takes the individual components in the assembly and the density map of the assembly as an input. The genetic algorithm starts with a population of assembly fits generated randomly using the feature points obtained through a vector quantization technique. The population of fits is iteratively improved through many generations by applying crossover and mutation operators and by retaining the best assembly fits based on the fitness function. The fittest member in the final generation is further refined using Flex-EM and produced as an output. The number of members N in the population is kept to 160 and the number of generations to 100.

Our goal is to identify a near-native configuration of a macromolecular assembly, given its individual protein components and a cryo-EM-derived density map at low to intermediate resolution. The predicted configuration needs to fit optimally into the density map, as well as satisfy the general physical rules of protein complexes, i.e. to avoid overlap between components. To this end, we adopted a GA that simultaneously fits the components into the density map. A GA works by discovering, emphasizing, and recombining good solutions in a highly parallel fashion, and is particularly suitable for solving computational problems that require searching through a huge number of possibilities for solutions (Mitchell, 1996). It starts with a set of candidate solutions and assumes that high-quality candidate “parent” solutions from different regions in the space can be combined to produce high-quality candidate “child” solutions. Our GA sampling scheme assumes no prior information about the starting positions and rotations of the assembly components in the map. The fitness function quantifies the match between the map and the model, and accounts for the atomic clashes between the components. We now describe the implementation details of the method (Figure 1).

### Sampling Using GA

#### Genotype Encoding

A “genotype” is made up of a number of variable entities called “genes.” These genes are the parameters that characterize the state of the model. A group of genotypes make up a “population” of assembly models. This population is iteratively improved upon, creating “generations” of new solutions and maintaining only the best scoring solutions in the population. In our assembly-fitting scenario, each genotype in the population describes the position and rotation of each component structure in the assembly map. Each genotype consists of two types of genes: a translation gene and a rotation gene (one each for every component). The translation gene is a 3D Cartesian vector representing the displacement of the component in Angstrom units (relative to an initial random position in the center of the map). The rotation gene is an integer indexing a list of quaternions.

#### Generation of Initial Population

The initial population is a set of randomly generated genotypes, or a set of genotypes seeded in some other fashion. Here, a vector quantization (VQ) algorithm was implemented to create a number of feature points in the target map that is equal to the number of assembly components (VQ feature-point set) (Zhang et al., 2010). The VQ algorithm uses a neural gas clustering technique to extract feature points from a density map following a procedure described elsewhere (Wriggers et al., 1999). Feature points are defined as the centers of density clusters, which as a whole capture the characteristic features of the density distribution. The result of the algorithm depends on the selected values for the density threshold (Zhang et al., 2010). Also, due to numerical instabilities, independent VQ runs with identical starting conditions can produce slightly different points. Since the variation for a given point position can only be up to 3 Å, for the purpose of GA we only use a set of VQ points produced from a single run using the density threshold value of 2σ from the mean. These feature points are assumed to roughly correspond to the centroids of each component. 50% of the genotypes in the initial population are created by randomly placing each component on any of the feature points. The remaining 50% are subjected to the same procedure, but an additional displacement is applied, with the maximum range equal to twice the minimum distance between all pairs of feature points. Orientations for each component are randomly selected from a uniform distribution of 5,000 quaternions (Shoemake, 1992). The population size is kept to 160.

#### Generation of New Population

New child genotypes constituting the new population are created using two different schemes, defined as crossover operations and diversity operations:

1. In the crossover operation, two genotypes are selected. This is done by applying a tournament selection (Mitchell, 1996) twice. This selection process starts by randomly picking two genotypes (tournament size = 2) from the population and by selecting only the one with the highest fitness genotype (parent). This process is repeated to produce two parent genotypes. For each gene in the fittest between the two selected parent genotypes, a crossover operation is applied by exchanging its value with the corresponding gene in the other parent. The modified genotype serves as the new child genotype. This operation is applied at a probability of 0.8. Each crossover event is followed by a mutation operation that randomly modifies the value of the crossed-over gene (in the child genotype). In a mutation operation, the translation gene is mutated by adding a random vector with a length ranging between 0 and the minimum of all the distances between the VQ points. The rotation gene is mutated by randomly replacing a quaternion (with equal probability for each quaternion). The probability of applying the mutation operation is typically set to 0.2 at the first generation and linearly decreases to 0.01 at the final generation. The crossover operation helps to create variation in the population, while the mutation operation is essential to avoid convergence to local minima.
2. In the diversity operation, two pairs of genes (each representing the state of a component defined by a translation and a rotation value) are randomly selected in the fittest genotype and swapped. A mutation operation (described above) is then applied to the child genotype. All genes in the child genotype are mutated with a constant mutation rate of 0.1. Child genotypes from the crossover and diversity operations constitute 90% and 10% of the total population size, respectively.

#### Selection Scheme and Termination

A new population consists of 160 child genotypes (same size as the initial population), which are created using crossover, diversity, and mutation operations, and are merged with the 160 genotypes from the previous parent generation. Then the 160 genotypes with the best fitness scores (see below) from the combined 320 child and parent genotypes are selected as the next-generation genotypes.

In our scheme, we run 20 independent GAs producing 20 predicted assembly fits (each starting from the same VQ point set). Each GA terminates after 100 generations. The output from each GA run is the predicted assembly that corresponds to the fittest genotype in the last generation.

### Fitness Function

The GA sampling is combined with a fitness function to quantify the match between the density map and the model (goodness of fit) as well as a clash score to prevent component volumes from overlapping with each other. The fitness score, *F*, is given by:

(Equation 1) *F* = (*n* × MI) − PS,

where *n* is the number of components in the assembly, MI is the mutual information representing the goodness of fit, and PS is a term to penalize for clashes.

The mutual information is calculated as follows (Farabella et al., 2015):

(Equation 2) $$\text{MI}\left(X;Y\right)=\sum_{x\in X}\sum_{y\in Y}p\left(x,y\right)\text{log}\frac{p\left(x,y\right)}{p\left(x\right)p\left(y\right)}\;,$$

where *X* and *Y* correspond to the density values of the voxels in the probe and target maps; *p*(*x*) and *p*(*y*) are given by the percentage of voxels with density values equal to *x* and *y*, respectively; and *p* (*x*,*y*) is given by the percentage of aligned voxels with value *x* in the probe map and *y* in the target map. The map density is divided into 20 bins. We have previously shown that this score performs well compared with the widely used cross-correlation coefficient (Vasishtan and Topf, 2011).

The PS is calculated by first generating for each component a grid with a value of 0 for all the volume elements (voxels). Then all voxels containing the backbone or the Cβ atoms of the components are set to a value of 1. For a given pair of grids, we calculate the ratio between the volume of the overlapping voxels and the sum of the volume of the voxels of the two individual grids (voxel size is set to 3.5 Å). The PS is defined as the sum of all pairwise fraction overlaps and can take any value greater than or equal to 0. The score was designed in such a way that severe atomic clashes between components are penalized while mild clashes are tolerated, to aid better sampling.

### Benchmark

**Figure 2:**
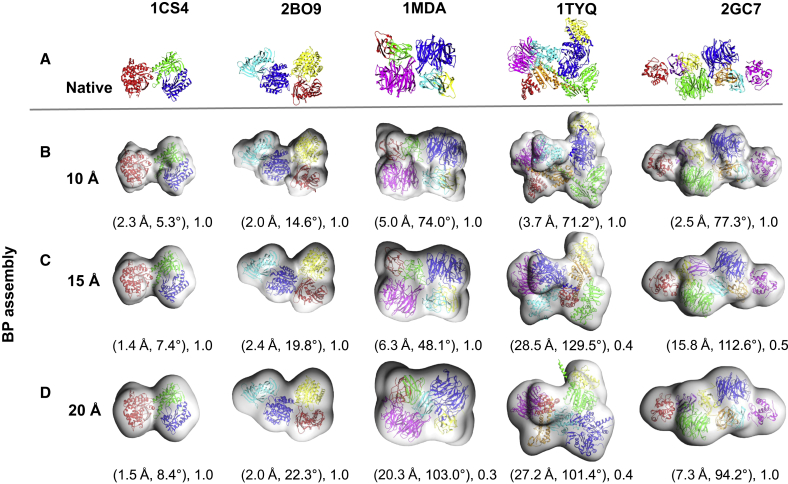
Representative Test Cases from the Simulated Benchmark (A) The structures of the native assemblies are shown with their PDB IDs. (B–D) The best-predicted (BP) assemblies found in the 20 GA runs using 10 Å (B), 15 Å (C), and 20 Å (D) simulated maps are shown below their corresponding native assemblies. The assembly placement score (APS) (translation in Angstroms and rotation in degrees) and the topology score (TS) are shown below each of the BP assemblies. Individual components of the assemblies are shown in cartoon representation with unique colors. The same coloring schemes are used for the individual components of the native and the predicted assemblies.

**Figure 3:**
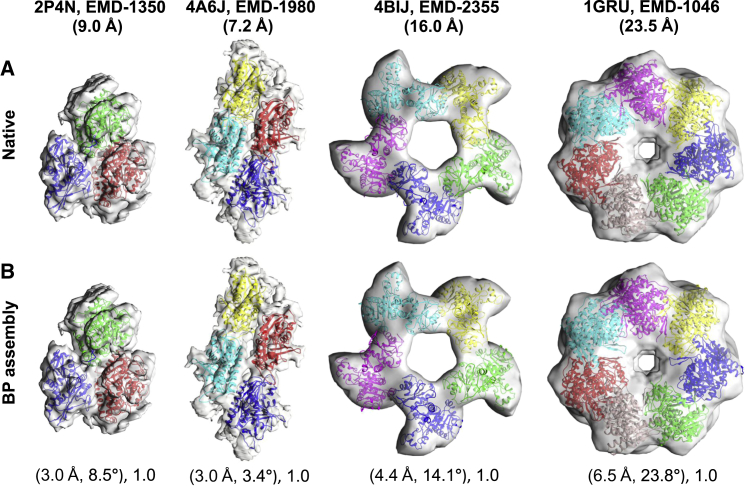
Representative Test Cases from the Experimental Benchmark (A) The experimental maps are shown with the associated fits (native). The PDB ID, EMD accession number, and resolution of the map are shown in the top row. (B) The BP assemblies found in the 20 GA runs are shown below their corresponding native assemblies. The APS (translation in Angstroms and rotation in degrees) and the TS are shown below each of the BP assemblies. Individual components of the assemblies are shown in cartoon representation with unique colors. The same coloring schemes are used for the individual components of the native and the predicted assemblies.

The method was tested on both simulated and experimental “target” maps of protein assemblies. The simulated benchmark contains a total of ten assemblies (Table 1). For each assembly, the method was tested using three different simulated maps at 10, 15, and 20 Å resolution. These maps were produced by blurring the atomic positions of the assemblies using a Gaussian point-spread function with sigma factor of 0.356 (Vasishtan and Topf, 2011). The voxel sizes of simulated maps were kept to 3.5 Å. The number of components in the assembly ranges from three to eight and the component size ranges between 88 and 525 residues. The experimental benchmark contains four assembly maps taken from the Electron Microscopy Databank (EMDB) (Lawson et al., 2011). The EMDB entries for the assembly maps are 1340, 1980, 2355, and 1046 at 9.0, 7.2, 16.0, and 23.5 Å resolution, respectively (Table 2). The PDB entries for the fits that correspond to EMDB maps are PDB: 2P4N, 4A6J, 4BIJ, and 1GRU, respectively. For measuring the prediction accuracy we consider a deposited fit as the reference fit (“native fit”). The number of components ranges from three to seven. Below, and in Tables 1 and 2, we describe the results of running γ-TEMPy for each of the test cases. For illustration purposes, we show in Figures 2 and 3 the results of five examples from the simulated benchmark (PDB: 1CS4, 2B09, 1MDA, 1TYQ, 2GC7, which represent different numbers of components) and all the test cases from the experimental benchmark, respectively.

### Prediction Accuracy: Simulated Benchmark

#### 10 Å Resolution

For the best-predicted (BP) assemblies using target maps simulated at 10 Å resolution, the topology score (TS, see Experimental Procedures for details) ranged between 0.8 and 1.0 (prior to refinement, Table 1A). The translation and rotation components of the assembly placement score (APS) (Lasker et al., 2009) (see Experimental Procedures for details) ranged from 1.3 to 7.9 Å and 13.3° to 79.9°, respectively (Table 1A). The Cα root-mean-square deviation (RMSD) (see Experimental Procedures for details) between the components of the BP assemblies and the corresponding native assemblies ranged from 3.2 to 16.9 Å (Table 1A). In eight of the ten cases (all except PDB: 1MDA and 1SGF), the BP assemblies identified by the GA had correct topology with TS = 1.0. In the case of PDB: 1MDA, only for chain M, the configuration deviated considerably with respect to the native assembly. The translation and rotation values of component placement score (CPS) were 19.6 Å and 82.2°, respectively (Table S1A). Similarly for the case of PDB: 1SGF, chain Y deviated considerably with respect to the native (CPS: translation = 27.8 Å and rotation = 81.4°) (Table S1A). In 50% of cases (PDB: 1CS4, 2DQJ, 2BO9, 1GPQ, and 2BBK), the topology of the highest-scoring (HS) assembly was correctly predicted, with a TS = 1.0, and in the case of PDB: 1CS4 it was also the BP assembly (Table 1A). The BP assembly was found within the top five ranks in eight of ten cases, and in seven of ten cases was found within top three ranks. In all the cases at 10 Å resolution, the fitness values of the native assemblies were always better than the predicted assemblies.

Following Flex-EM refinement (Topf et al., 2008) (see Experimental Procedures for details), the APS for the BP assemblies ranged from 0.5 to 8.2 Å for the translational and 1.0° to 77.3° for the rotational score components, respectively (Figure 2A and Table 1A). The refinement helped to reduce the RMSD of the BP assembly of PDB: 1CS4 from 4.0 to 2.8 Å; of PDB: 2DQJ from 3.5 to 0.7 Å; of PDB: 1VCB from 7.7 to 4.8 Å; and of 1GPQ from 3.2 to 0.6 Å (Table 1A). In all other cases (four of which had TS = 1 prior to refinement), the refinement resulted in a marginal decrease or increase in RMSD. This is due to the fact that in those cases the starting fits (BP) before refinement deviate (at least in one of the components) considerably from the native structure with a minimum and maximum RMSD of 10.9 and 16.9 Å, respectively. It is worth noting that for the case of PDB: 1MDA (a six-component assembly), the TS improved from 0.8 to 1.0 after Flex-EM refinement (Table 1A). Despite this improvement, the RMSD indicated that the model is far from its native configuration (reduced from 14.1 to 12.0 Å). The Cα RMSD with respect to the native component chain IDs J, H, and L was 4.8, 7.3, and 2.9 Å, respectively, whereas the Cα RMSD of the components with chain IDs M, B, and A was 16.5, 22.9, and 17.6 Å, respectively. The CPS (the translation and rotation pair) for the latter three chains was (9.7 Å, 86.9°), (8.1 Å, 155.6°), and (2.0 Å, 157.1°), respectively. Even though all the components were placed correctly, as evidenced by the good TS, the higher RMSD for chains M, B, and A resulted mainly from a rotation of these chains relative to their corresponding native position (Figure 2B, chains M, B, and A shown in green, red, and yellow, respectively).

#### 15 Å Resolution

For the BP assemblies calculated using target maps simulated at 15 Å resolution, the TS ranged between 0.4 and 1.0 (prior to refinement, Table 1B). The value of the translation and the rotation components of the APS ranged from 1.9 to 28.2 Å and 8.8° to 126.4°, respectively. The RMSD of the components of the BP assemblies ranged from 2.9 to 39.0 Å (Table 1B). In seven of the ten cases, the BP assemblies identified by the GA had correct topology (TS = 1.0, Table 1B). For the remaining three cases, the CPS revealed that only the chain IDs of PDB: 1SGF (Y: 21.5 Å, 38.8°), 1TYQ (A: 47.7 Å, 128.2°; B: 45.0 Å, 158.4°; F: 46.9 Å, 153.0°; G: 46.4 Å, 102.3°) and 2GC7 (F: 23.3 Å, 75.4°; B: 18.0 Å, 149.3°; C: 36.4 Å, 150.6°; G: 29.8 Å, 154.2°) deviated considerably with respect to the native (Table S1C). Similarly to the 10 Å case, in 50% of the examples (PDB: 1CS4, 2DQJ, 2BO9, 1GPQ, and 2BBK) the topology of the HS assemblies was correctly predicted, and in the case of PDB: 2DQJ it was also the BP assembly (Table 1B). The BP assembly was found within the top five ranks in six out of ten cases and in the top ten in eight of the ten cases. For PDB: 2DQJ (for which the BP assembly is the same as the HS assembly), notable improvement was observed after Flex-EM refinement, with a decrease of RMSD from 2.9 to 0.7 Å (Table 1B). In all the cases at 15 Å resolution, the fitness values of the native assemblies were always better than the predicted assemblies.

Following refinement, the value of the translation and the rotation components of the APS for the BP assemblies ranged from 0.6 to 28.5 Å and 1.7° to 129.5°, respectively (Figure 2B and Table 1B). The RMSD from the native was reduced from 3.0 to 2.3 Å for PDB: 1CS4, from 2.9 to 0.7 Å for PDB: 2DQJ, from 17.0 to 14.2 Å for PDB: 1VCB, from 6.1 to 5.4 for PDB: 2BO9, and from 17.9 to 16.6 Å for PDB: 1SGF (Table 1B).

#### 20 Å Resolution

For the BP assemblies calculated using target maps simulated at 20 Å resolution, the TS ranged between 0.3 and 1.0 (prior to refinement, Table 1C). The value of the translation and the rotation components of the APS ranged from 1.5 to 27.1 Å and 13.9 to 101.5°, respectively (Table 1C). The RMSD of the BP assemblies ranged from 3.4 to 36.0 Å (Table 1C). In six of the ten cases, the BP assemblies identified by the GA had correct topology (TS = 1.0). For the other four cases the CPS revealed that the chain IDs of PDB: 1VCB (B: 33.2 Å, 95.1° and C: 21.7 Å, 89.6°), PDB: 1MDA (M: 23.1 Å, 122.9°; L: 12.0 Å, 130.6°; B: 32.5 Å, 125.8°; A: 37.3 Å, 114.0°), PDB: 1SGF (Y: 19.5 Å, 161.0°), and PDB: 1TYQ (A: 33.0 Å, 97.3°; B: 37.8 Å, 97.5°; C: 38.9 Å, 86.4°; F: 21.4 Å, 124.7°; G: 51.0 Å, 178.1°) deviated considerably with respect to the native (Table S1E). In four of the ten cases (PDB: 2DQJ, 2BO9, 1GPQ, and 2BBK) the HS assemblies had the correct topology, and in the case of PDB: 2DQJ it was also the BP assembly (Table 1C). The BP assembly was found within the top five ranks in four out of ten cases and in the top ten in six of the ten cases. In all the cases at 20 Å resolution, the fitness values of the native assemblies were always better than the predicted assemblies.

Following Flex-EM refinement, the value of the translation and the rotation components of the APS for the BP assemblies ranged from 0.6 to 27.2 Å and 1.9° to 103.0°, respectively (Figure 2C and Table 1C). The RMSD of the BP assemblies PDB: 1CS4 and 2DQJ with respect to their corresponding native structures was reduced significantly, from 5.0 to 2.6 Å and from 3.4 to 0.7 Å, respectively (Table 1C).

### Prediction Accuracy: Experimental Benchmark

Using experimental target maps, the TS of the BP and HS assemblies for all four cases were found to be 1.0 (prior to refinement, Table 2). The value of the translation and the rotation components of the APS ranged from 4.3 to 11.3 Å and 14.6° to 21.0°, respectively (Table 2). In the case of the HS assembly of PDB: 2P4N, only for chain A, the configuration considerably deviated with respect to the native assembly. The translation and rotation values of the CPS were 5.2 Å and 153.1°, respectively (Table S2B). The RMSD of the BP assemblies ranged from 6.3 to 13.7 Å (Table 2). The BP assembly was found within the top five ranks in all four cases. It is also worth noting that, for the symmetrical cases PDB: 4BIJ and 1GRU, the method identified near-native topologies for both the BP and HS assemblies without the use of symmetry restraints. In all of these cases, the fitness values of the native assemblies are always better than the predicted assemblies.

Following Flex-EM refinement, the value of the translation and the rotation components of the APS for the BP assemblies ranged from 3.0 to 6.5 Å and 3.4° to 23.8°, respectively (Table 2 and Figure 3). The RMSD of the BP assemblies was reduced in all cases: from 6.7 to 4.0 Å for PDB: 2P4N; from 6.3 to 3.2 Å for PDB: 4A6J; from 13.7 to 6.8 Å for PDB: 4BIJ; and from 11.7 to 10.6 Å for PDB: 1GRU (Table 2).

### Effects of VQ Feature Points on Prediction Accuracy

Each genotype (representing an assembly) in the initial population is randomized based on the VQ feature-point set (see Theory). To assess the effect of feature-point quality on our results, we calculated the similarity between each VQ feature-point set (of each test case in our benchmarks) and the point sets representing the component centroids calculated from the corresponding native assembly (centroid point set). To this end, we used the Hausdorff distance (HD) metric (Huttenlocher et al., 1993). Given two finite point sets A and B, the HD determines the degree of resemblance between them as follows:

(Equation 3) HD(A,B)=max(h(A,B),h(B,A)),

where

(Equation 4) $$h\left(A,B\right)=\underset{a\in A}{\text{max}}\underset{b\in B}{\text{min}}d\left(a,b\right)$$

and *d*(*a,b*) is the Euclidean distance between points *a* and *b*. Identical point sets will have HD = 0, and the HD will increase with increasing dissimilarity.

**Figure 4:**
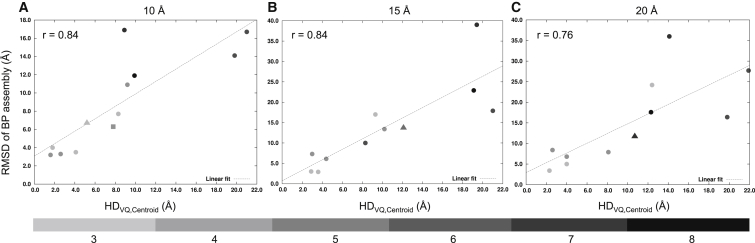
Effect of Feature-Point Set Generation by Vector Quantization on Prediction Accuracy (A–C) The linear relationship between the average Cα RMSD of the BP assembly and HD_VQ,centroid_ (Hausdorff distance between the VQ points set of the density map and the point set calculated from the centroids of the native assembly components) is shown for 10 Å (A), 15 Å (B), and 20 Å (C) resolution maps. Data points for the experimental cases PDB: 2P4N (resolution = 9.0 Å, shown as filled triangle) and PDB: 4A6J (resolution = 7.2 Å, shown as filled square) have been added to (A). The data points for the experimental cases PDB: 4BIJ (resolution = 16.0 Å) and PDB: 1GRU (resolution = 23.5 Å), both shown as filled triangles, have been added to (B) and (C), respectively. The best-fitting regression line (linear fit) along with the Pearson's correlation coefficient (r) are indicated on the plots. The gray data points are values based on the number of components in the assembly using a gray-scale gradient (shown at the bottom of the figure). See also Figure S1.

Figure 4 shows that the relationship between the RMSD of the BP assembly from the native assembly (before refinement) and the HD between the VQ point set and the centroid point set (HD_VQ,centroid_) is linearly correlated in all three resolutions, with Pearson's correlation coefficient of 0.84, 0.84, and 0.76 for 10, 15, and 20 Å resolution maps, respectively. Therefore, the ability of the GA to identify near-native assemblies decreases with increasing deviation between the native centroid point set and the VQ point set (and this problem is likely to worsen with an increasing number of components). In all cases where the HD between the VQ points and the native centroid point set is less than or equal to 5 Å, the RMSD of the BP assemblies was between 2.9 and 8.4 Å.

We next compared the feature points obtained by our VQ method and GMFIT, which is based on Gaussian mixture models (GMM) (Kawabata, 2008) (Figure S1). For GMFIT (as in our VQ implementation) the number of feature points calculated was set to the number of components in the assembly. The Pearson's correlation coefficient between HD_VQ,centroid_ and HD_GMFIT,centroid_ (HD between the GMFIT point set and centroid point set) was 0.44, 0.75, and 0.67 at resolutions 10, 15, and 20 Å, respectively, showing that there is less agreement between the two methods at 10 Å than at worse resolutions. The average HD_VQ,centroid_ at 10, 15, and 20 Å resolution was 8.4, 10.3, and 10.2 Å, respectively, and the average HD_GMFIT,centroid_ was 9.2, 11.9, and 10.8 Å, respectively.

**Figure 5:**
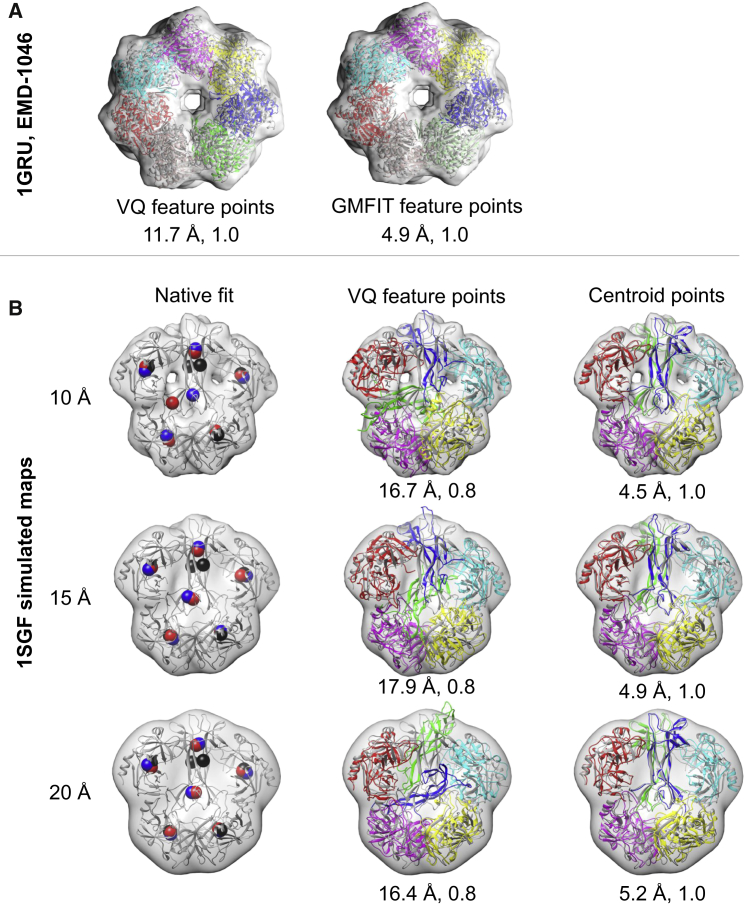
Effect of Feature-Point Set Generation by Different Methods on Prediction Accuracy (A) The BP assembly is shown for the experimental case, PDB: 1GRU using VQ (left) and GMFIT (right) feature-point set generated from the density map at 23.5 Å resolution (EMD-1046). The BP assembly obtained using GMFIT feature points is also the highest-scoring assembly. The native fit (associated with the map) is colored in gray and the components of the predicted assemblies are colored uniquely. The values of the average Cα RMSD from the native assembly and the TS are shown at the bottom of the respective predictions. (B) The first column shows the feature points (as spheres) obtained using the centroids of the individual components of the assembly PDB: 1SGF (black), VQ (blue), and GMFIT (red) for the simulated maps at resolution 10, 15, and 20 Å. The native assemblies corresponding to the simulated maps are shown as cartoons and colored in gray. The second column shows the BP assembly by the GA for the simulated maps at resolution 10, 15, and 20 Å using the VQ-based feature points. The third column shows the BP (in this case, the BP assembly is the HS assemblies) predicted by the GA for the simulated maps at resolution 10, 15, and 20 Å using the centroid-based feature points. The native assembly corresponding to the simulated maps is colored gray and the components of the predicted assemblies are colored uniquely. The values of the average Cα RMSD from the native assembly and the TS are shown at the bottom of the respective predictions.

Given the variations between the feature-point set obtained by different methods, we expected that the likelihood of obtaining better predictions could potentially be improved by using multiple methods. To test this hypothesis, we ran the GA using the feature-point set generated by GMFIT for the PDB: 1GRU case (GroEL), with the experimental map (EMD-1046, Table 2) at resolution 23.5 Å. For this case, GMFIT approximated the positions of the components of the native assembly better than VQ (HD_VQ,centroid_ = 10.7 Å and HD_GMFIT,centroid_ = 1.4 Å). After running 20 GA predictions, the RMSD of both the HS and BP assemblies was 4.9 Å, in comparison with 13.2 Å and 11.7 Å, respectively, for our original prediction using VQ feature points (Figure 5A).

Next we examined a specific case, PDB: 1SGF, whereby both methods performed badly (with high variation between the feature points and the native centroids), resulting in bad assembly predictions by the GA. In this case, HD_VQ,centroid_ was 21.0, 21.0, and 19.8 Å and HD_GMFIT,centroid_ was 18.7, 20.0, and 20.1 Å at 10, 15, and 20 Å resolution, respectively. Further analysis showed that the feature points calculated by both methods approximated correctly the positions of the centroids in four chains (A, G, X, and Z) for all three simulated resolutions (Figure 5B, top panel). However, for the two remaining chains (B and Y, which are elongated and closely packed relative to the other components in the assembly), the VQ feature points did not approximate the corresponding native centroid positions. The RMSD of the BP assembly starting with VQ feature points (before Flex-EM refinement) was 16.7, 17.9, and 16.4 Å at 10, 15, and 20 Å resolution (Table 1). As a control experiment, we ran 20 GA runs for PDB: 1SGF, starting with feature points calculated from the centroid positions of the native components. The results improved considerably (without Flex-EM refinement), with the HS (and the identical BP) assembly having RMSD of 4.5, 4.9, and 5.2 Å for 10, 15, and 20 Å resolution, respectively (Figure 5B, bottom panel).

### Effect of Resolution on Prediction Accuracy

From the above results we found that the accuracy of the method depends strongly on the accuracy of the initial feature points. To test the effect of map resolution on the prediction accuracy, we ran 20 GAs on the simulated benchmark at 10 and 20 Å resolution considering the native centroids of the assembly components as the starting positions.

For 10 Å resolution, the method was able to sample the native configuration for all test cases (based on TS) (Table S3). For the BP assembly, the value of the APS ranged from 0.1 to 0.4 Å (translation) and 7.7° to 49.6° (rotation), respectively (Table S3A). The RMSD of the BP assemblies ranged from 1.8 to 9.1 Å (Table S3A). In nine out of ten cases, the RMSD of the BP assemblies was <5 Å. In all cases, the BP assemblies identified by the GA had correct topology (TS = 1.0), and the HS assemblies identified also had correct topology (Table S3A). The BP assembly was found within the top five ranks in nine of the ten cases.

At 20 Å resolution the performance did not deteriorate significantly, with the method sampling the native configuration for all test cases (Table S4). For the BP assembly, the value of the translation and the rotation components of the APS ranged from 0.1 to 0.4 Å and 7.0° to 77.4°, respectively (Table S4A). The RMSD of the BP assemblies ranged from 1.7 to 11.4 Å (Table S4A). In eight out of ten cases, the RMSD of the BP assemblies was approximately <5 Å. In all cases, the BP assemblies identified by the GA had correct topology (TS = 1.0) and in all except PDB: 2GC7, the identified HS assemblies also had correct topology (Table S4A). The BP assembly was found within the top five ranks in nine of the ten cases.

In general, given a better guess for the initial feature points, the method is able to efficiently sample and rank the near-native topologies at 10 and 20 Å resolutions, and is not significantly affected by the difference in resolutions. For the ten cases considered in the benchmark, the average translation and rotation components of the APS for 10 Å resolution was 0.1 Å and 15.4°, respectively. The average translation and rotation components for 20 Å resolution was 0.2 Å and 24.8°, respectively. The results did, however, show more accurate predictions for higher-resolution maps in terms of the orientation of the components (especially in large assemblies containing globular components). For example, in the case of PDB: 2GC7 at 20 Å resolution, a near-native topology was obtained for the BP assembly. However, the CPS score shows a large rotation of chains C (170.0°), F (167.7°), and G (164.8°) with respect to their position in native assembly (Table S4B) compared with the results obtained using 10 Å resolution (6.2°, 13.7°, and 13.0°, respectively; Table S3B).

### GA Convergence and Computation Time

**Figure 6:**
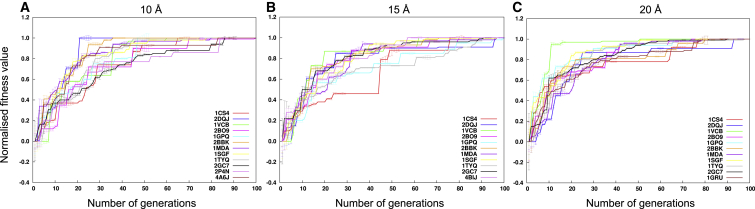
Fitness Value Profile The value of the fitness function for the fittest member in the population during the GA generations is shown for 10 Å (A), 15 Å (B), and 20 Å (C) resolution maps. The fitness profile of the experimental cases PDB: 2P4N (resolution = 9.0 Å) and PDB: 4A6J (resolution = 7.2 Å) has been added to (A). The fitness profiles of the experimental case PDB: 4BIJ (resolution = 16.0 Å) and PDB: 1GRU (resolution = 23.5 Å) have been added to (B) and (C), respectively. The profile for each case is colored uniquely. The length of the error bar shown in each profile equals the SD of the fitness values of the population in any given generation. The fitness values shown are normalized between 0 and 1 and the number of generations runs from 1 to 100. See also Figure S2.

The convergence of the GA is observed by plotting the value of the fitness function of the fittest member (assembly configuration) in the population at each of the 100 generations, along with the variation in the population (Figure 6). On average, the GA converged within 68, 83, and 82 generations at 10, 15, and 20 Å resolution, respectively (including the experimental cases in Figure 6A–C). The results suggest that at a higher resolution (10 Å) the convergence is typically faster, most likely due to the fact that the scoring function has more discriminatory power.

CPU times for the 20 GA runs on the experimental benchmark and the simulated 20 Å resolution maps was recorded (Figure S2). For every generation, the fitness function value of all the members in the population (of size 160) was calculated in parallel using 40 processing units (four members per processor). All the calculations were performed on 2.6-GHz AMD processors. We found a strong linear correlation (0.92) between the number of voxels in a given density map and the processing time. In addition, the running time will also scale with the number of generations and population size used. In this study the number of generations and the population size was fixed for the whole benchmark. All other parameters do not affect the running time. The minimum, maximum, and average processing time to generate 20 GA predictions (including the 20-Å simulated and experimental benchmark) was ∼4, 49, and 17 hr, respectively, and on average, one GA prediction takes about 50 min to complete.

## Discussion

To better interpret 3D EM maps of large macromolecular assemblies, in particular at low to intermediate resolutions, we have developed a method for simultaneous density fitting of multiple assembly components. To address such a complex optimization problem (with a search space that exponentially increases in relation to the number of components), only a handful of approaches have so far been developed with the EM density being the only experimental information used (Kawabata, 2008, Lasker et al., 2009, Lasker et al., 2010, Zhang et al., 2010, Rusu and Birmanns, 2010, Esquivel-Rodriguez and Kihara, 2012). Our method relies on a GA to efficiently identify optimal solutions to the problem and, to our knowledge, is the first method to apply the mutual information as the goodness-of-fit score within the context of assembly fitting. Based on the benchmark, we have tested and identified optimum values for the GA parameters including the size of the population, number of generations, and crossover and mutation rates. Given these parameters, we demonstrated that the use of a simple clash penalty score in a weighted combination with the goodness-of-fit score was sufficient to guide the sampling and identify correct native topology. However, the method is, in principle, flexible, and the user can modify the various parameters to suite a specific case. In general, larger complexes (number of components >8) may require bigger population sizes (>160) and generations (>100). We also showed that predicted assemblies with an approximate RMSD <5 Å from the corresponding native assembly can be further improved with Flex-EM refinement. Naturally, the method has been more successful with a lower number of components (three or four), but it has been shown to identify correct configuration fits even with assemblies containing as many as eight components using a 20 Å resolution map.

The potential energy landscape underlining the assembly-fitting problem is very complex. To efficiently sample the huge configurational space, we designed the method to focus the search around the density feature points derived from the map. Hence, the quality of the density feature points is crucial for the success of the method. In this study we used a VQ technique to derive density feature points from the map. Our method was found to depend strongly on the density feature points used as input. However, generating feature points that accurately represent the native centroids of the assembly components can be very challenging when proteins have an elongated or narrow shape or are closely packed in the assembly (e.g. in the case of PDB: 1SGF). This issue has been observed in the problem of density map segmentation (Pintilie et al., 2010). As a proof of principle, we have shown that the accuracy of the GA prediction tends to improve by using better approximations for the feature points (here obtained using GMM). The limitation of the method in identifying very accurate initial feature points could be made less critical by, for example, running multiple independent GA predictions using different feature-point sets (obtained by different techniques) as well as crosstalk between independent GA runs (to better explore the search space). These variable feature points may help the GA to sample the new regions of the conformational space and thus improve the likelihood of obtaining native-like assembly fits. In fact, assuming the native centroids of the assembly components as the starting points, the method was able to find the native topologies for all the simulated benchmarks at 10 and 20 Å resolution, with RMSD of the BP assemblies less than ∼5 Å in more than 80% of the cases.

Since the GA is non-deterministic, it is impossible to predict the running time necessary to definitely produce a perfect solution. However, a trade-off can be achieved between a quicker run time for less accurate results and a longer run time for more accurate results by adjusting the population size and the number of generations. A lower value for the population size and the number of generations will produce quicker and less accurate results. To further optimize the position and orientation of the components, we used Flex-EM real-space refinement. The refinement showed improvement for most of the fits predicted, with average Cα RMSD less than or equal to ∼5 Å from the native assembly, but failed to improve fits that were correctly placed (based on TS) but oriented significantly differently from the native (e.g. the case of PDB: 1MDA at 10 Å resolution). In the future, the method will incorporate component flexibility to better interpret the conformational difference between the complex map and the individual components of the assembly as well as partial fitting (if not all components are known). Additional improvements could potentially be achieved by adding spatial restraints from other experimental data (Amir et al., 2015, Russel et al., 2012, van Zundert et al., 2015).

## Experimental Procedures

### Refinement Using Flex-EM

To explore the possibility of further improving the results we added a refinement step, which was applied only to the “best solutions.” From the prediction of 20 independent GA solutions we define two best solutions, namely, the best-predicted assembly (BP, the assembly with the lowest Cα RMSD from the native) and the highest-scoring assembly (HS, the assembly with the highest fitness score). The BP and HS assemblies are subjected to a refinement using Flex-EM (Topf et al., 2008). Each component in the assembly was considered as a rigid body during the refinement. The number of MD cycles was kept to five for the simulated benchmark. For the experimental benchmark, the number of Flex-EM refinement cycles was ten, because the number of residues in those assemblies was approximately 2-fold larger than the simulated benchmark.

### Measures of Model Accuracy

The accuracy of the predictions was reported using the following three metrics.

#### Topology Score

The Topology Score (TS) indicates the fraction of components that are positioned correctly. We first define a sphere around each component in the native assembly. The center of the sphere is set to the center of mass (COM) of the component. The radius of the sphere is set to the radius of gyration of the component. We then consider a predicted component to be placed correctly if its COM falls within its corresponding sphere of the native component.

#### Placement Scores

The accuracy of the position and rotation of each predicted component was also calculated using the Component Placement Score (CPS, originally called OS score) that describes the translation (in Angstroms) and the rotation angle (in degrees) needed to superpose the predicted component onto the corresponding native component (Lasker et al., 2009, Topf et al., 2008). The Assembly Placement Score (APS) was defined as the average of all of the CPS scores in the predicted assembly (Lasker et al., 2009).

#### RMSD

We calculated the average of the individual root-mean-square deviation (RMSD) of the Cα atom positions of each component in the predicted assembly from the corresponding Cα atom positions in the native component. For assemblies with two or more identical components, we identified the correspondence between the predicted and the native component that gave the minimum average Cα RMSD.

## Author Contributions

Conceptualization: A.P.P., D.V., F.A., and M.T. Methodology: A.P.P., D.V., F.A., and M.T. Investigation: A.P.P. and M.T. Writing, original draft: A.P.P. and M.T. Writing, review and editing: A.P.P., D.V., F.A., and M.T. Funding acquisition: F.A. and M.T.

## Figures and Tables

**Table 1:** Summary of Model Accuracy in the Simulated Benchmark

NC	Test Case^a^	BP			BP after Flex-EM			HS			HS after Flex-EM			Rank of BP
TS	APS (Å, °)	RMSD (Å)	TS	APS (Å, °)	RMSD (Å)	TS	APS (Å, °)	RMSD (Å)	TS	APS (Å, °)	RMSD (Å)
**A. 10 Å Resolution**														

3	1CS4	1.0	1.4, 15.0	4.0	1.0	2.3, 5.3	2.8	1.0	1.4, 15.0	4.0	1.0	2.3, 5.3	2.8	1
2DQJ	1.0	2.3, 15.5	3.5	1.0	0.6, 1.5	0.7	1.0	2.3, 18.0	3.9	1.0	0.7, 8.7	1.7	5
1VCB	1.0	5.3, 28.4	7.7	1.0	2.5, 21.8	4.8	0.3	19.0, 91.3	25.3	0.3	19.1, 91.8	25.5	16
4	2BO9	1.0	1.5, 13.3	3.3	1.0	2.0, 14.6	4.0	1.0	1.4, 49.6	9.9	1.0	2.5, 50.2	10.3	2
1GPQ	1.0	1.3, 15.4	3.2	1.0	0.5, 1.0	0.6	1.0	1.4, 16.4	3.3	1.0	1.1, 21.7	3.9	3
2BBK	1.0	5.6, 54.9	10.9	1.0	3.9, 61.3	11.3	1.0	5.4, 89.6	14.9	1.0	5.9, 82.8	15.3	2
6	1MDA^b^	0.8	7.7, 79.9	14.1	1.0	5.0, 74.0	12.0	0.8	7.7, 79.9	14.1	1.0	5.0, 74.0	12.0	1
1SGF	0.8	7.9, 58.7	16.7	0.8	8.2, 58.3	16.1	0.8	8.0, 87.4	20.5	0.8	9.6, 89.9	19.7	9
7	1TYQ^b^	1.0	4.6, 71.2	16.9	1.0	3.7, 71.2	16.3	0.7	24.8, 87.8	34.8	0.7	27.8, 89.8	37.7	3
8	2GC7^b^	1.0	4.6, 77.1	11.9	1.0	2.5, 77.3	10.9	0.6	16.5, 88.4	22.5	0.6	16.7, 89.3	23.3	2

**B. 15 Å Resolution**														

3	1CS4	1.0	1.9, 8.8	3.0	1.0	1.4, 7.4	2.3	1.0	1.9, 10.0	3.1	1.0	0.8, 1.5	0.9	2
2DQJ	1.0	2.1, 10.0	2.9	1.0	0.6, 1.7	0.7	1.0	2.1, 10.0	2.9	1.0	0.6, 1.7	0.7	1
1VCB	1.0	6.2, 116.8	17.0	1.0	3.9, 101.5	14.2	0.0	27.7, 122.2	35.7	0.0	28.3, 106.7	35.7	20
4	2BO9	1.0	3.5, 21.5	6.1	1.0	2.4, 19.8	5.4	1.0	3.6, 49.4	11.4	1.0	2.4, 51.2	10.7	4
1GPQ	1.0	2.9, 37.5	7.3	1.0	3.2, 40.2	7.8	1.0	2.9, 90.6	12.8	1.0	3.2, 87.2	12.7	8
2BBK	1.0	7.1, 57.5	13.4	1.0	5.8, 65.5	13.4	1.0	6.9, 157.8	29.8	1.0	9.2, 146.0	29.6	12
6	1MDA^b^	1.0	5.5, 50.0	10.0	1.0	6.3, 48.1	10.3	0.7	22.9, 95.2	28.1	0.7	22.8, 93.1	28.4	7
1SGF	0.8	6.8, 75.7	17.9	0.8	6.5, 77.5	16.6	0.8	6.8, 88.2	20.3	0.8	6.2, 89.0	19.6	2
7	1TYQ^b^	0.4	28.2, 126.4	39.0	0.4	28.5, 129.5	39.6	0.4	37.5, 79.1	42.4	0.4	39.9, 83.1	45.5	5
8	2GC7^b^	0.5	15.1, 101.4	22.9	0.5	15.8, 112.6	24.5	0.5	15.1, 101.4	22.9	0.5	15.8, 112.6	24.5	1

**C. 20 Å Resolution**														

3	1CS4	1.0	3.2, 13.9	5.0	1.0	1.5, 8.4	2.6	0.3	17.8, 103.7	25.4	0.3	18.1, 99.4	25.6	4
2DQJ	1.0	1.5, 16.8	3.4	1.0	0.6, 1.9	0.7	1.0	1.5, 16.8	3.4	1.0	0.6, 1.9	0.7	1
1VCB	0.3	19.7, 72.6	24.2	0.3	17.4, 77.5	23.8	0.3	31.5, 163.1	39.5	0.0	31.9, 160.9	39.1	12
4	2BO9	1.0	2.7, 26.9	6.8	1.0	2.0, 22.3	5.7	1.0	2.6, 50.3	10.4	1.0	2.2, 50.8	10.6	12
1GPQ	1.0	2.2, 52.2	8.4	1.0	2.3, 53.8	9.0	1.0	2.2, 121.0	18.0	1.0	2.2, 117.2	17.7	2
2BBK	1.0	5.5, 26.0	7.9	1.0	6.4, 30.0	9.9	1.0	5.3, 56.1	11.8	1.0	5.6, 63.9	12.9	3
6	1MDA^b^	0.5	19.2, 101.5	27.7	0.3	20.3, 103.0	28.5	0.7	20.0, 124.6	29.6	0.5	20.4, 125.2	30.2	12
1SGF	0.8	6.6, 86.9	16.4	0.8	6.9, 90.0	16.6	0.8	6.9, 92.1	18.6	1.0	7.1, 95.2	18.0	14
7	1TYQ^b^	0.3	27.1, 99.2	36.0	0.4	27.2, 101.4	36.6	0.1	47.8, 142.1	58.1	0.1	50.1, 143.4	59.6	8
8	2GC7^b^	1.0	6.9, 97.9	17.6	1.0	7.3, 94.2	17.6	0.8	12.4, 138.5	26.6	0.8	14.6, 132.8	27.4	6

NC, the number of components in the assembly; Test case, the PDB ID of the assemblies; BP, the best-predicted assembly with the lowest average Cα RMSD from the native among 20 GA runs; BP after Flex-EM, the BP assembly obtained after performing Flex-EM refinement; HS, the highest-scoring assembly among 20 GA runs; HS after Flex-EM, the HS assembly obtained after performing Flex-EM refinement; TS, the topology score describing the fraction of components placed correctly; APS, the assembly placement score describing the average shift in Angstroms and rotation in degrees needed to superpose all the predicted components onto their corresponding native components; RMSD, the average Cα RMSD in Angstroms between the predicted components and its corresponding native components; Rank of BP, the rank of the BP among 20 GA predictions based on the fitness function value. See also Table S1.

a Test cases PDB: 1CS4, 2DQJ, and 1VCB represent asymmetric complexes. For the test cases PDB: 1GPQ and 2BBK, the biological unit is a tetramer with C2 symmetry. For the test cases PDB: 1MDA and 1SGF, the biological unit is a hexamer with C2 symmetry. For PDB: 2GC7, the biological unit is a tetramer with the asymmetric unit containing four biological units. For the purpose of the benchmark, we used two biological units, which has a total of eight components.

b N-terminal residues have been removed in: PDB: 1MDA chains H and J (1–31), 2GC7 chains A and E (5–44), and PDB: 1TYQ chain G (11–27).

**Table 2:** Summary of Model Accuracy in the Experimental Benchmark

EMDB ID	Fitted PDB ID	NC	Resolution (Å)	Voxel Size (Å)	BP			BP after Flex-EM			HS			HS after Flex-EM			Rank of BP
TS	APS (Å, °)	RMSD (Å)	TS	APS (Å, °)	RMSD (Å)	TS	APS (Å, °)	RMSD (Å)	TS	APS (Å, °)	RMSD (Å)
1340	2P4N^a^	3	9.0	2.0	1.0	4.3, 18.3	6.7	1.0	3.0, 8.5	4.0	1.0	4.3, 60.2	13.3	1.0	3.7, 56.0	12.4	3
1980	4A6J^b^	4	7.2	1.6	1.0	4.5, 14.6	6.3	1.0	3.0, 3.4	3.2	1.0	4.5, 14.6	6.3	1.0	3.0, 3.4	3.2	1
2355	4BIJ^c^	5	16.0	4.4	1.0	11.3, 19.9	13.7	1.0	4.4, 14.1	6.8	1.0	11.3, 19.9	13.7	1.0	4.4, 14.1	6.8	1
1046	1GRU^d^	7	23.5	2.8	1.0	8.9, 21.0	11.7	1.0	6.5, 23.8	10.6	1.0	9.0, 27.8	13.2	1.0	8.3, 26.2	12.2	5

EMDB ID, electron microscopy databank ID of the experimental map of the assembly; Fitted PDB ID, the PDB ID of fit associated with the experimental map; NC, the number of components in the assembly; Resolution, the resolution of the map in Angstroms; Voxel size, the size of the grid spacing of the map along all three axes. Other definitions as for Table 1. See also Table S2.

a Test case PDB: 2P4N represents an asymmetric complex.

b The biological unit of PDB: 4A6J is a decamer with helical symmetry. For the purpose of the benchmark, we used only four components.

c The biological unit of PDB: 4BIJ is a pentamer with C5 symmetry.

d The biological unit of GroEL PDB: 1GRU is a hetero 21-mer with C7 symmetry. For the purpose of the benchmark, we only used one GroEL ring containing seven components.

## Supplemental Information

Document S1. Figures S1 and S2, and Tables S1–S4

Document S2. Article plus Supplemental Information
