# Supplementary material for: γ-TEMPy: Simultaneous Fitting of Components in 3D-EM Maps of Their Assembly Using a Genetic Algorithm
Source: Structure. 2015 Dec 1;23(12):2365–76. doi: 10.1016/j.str.2015.10.013 (PMC4671957; doi:10.1016/j.str.2015.10.013)
Supplement: Document S1. Figures S1 and S2, and Tables S1–S4 [file mmc1.pdf]

Structure, Volume *23*

Supplemental Information

γ-TEMPy: Simultaneous Fitting of Components in 3D-EM Maps of Their Assembly Using a Genetic Algorithm

Arun Prasad Pandurangan, Daven Vasishtan, Frank Alber, and Maya Topf

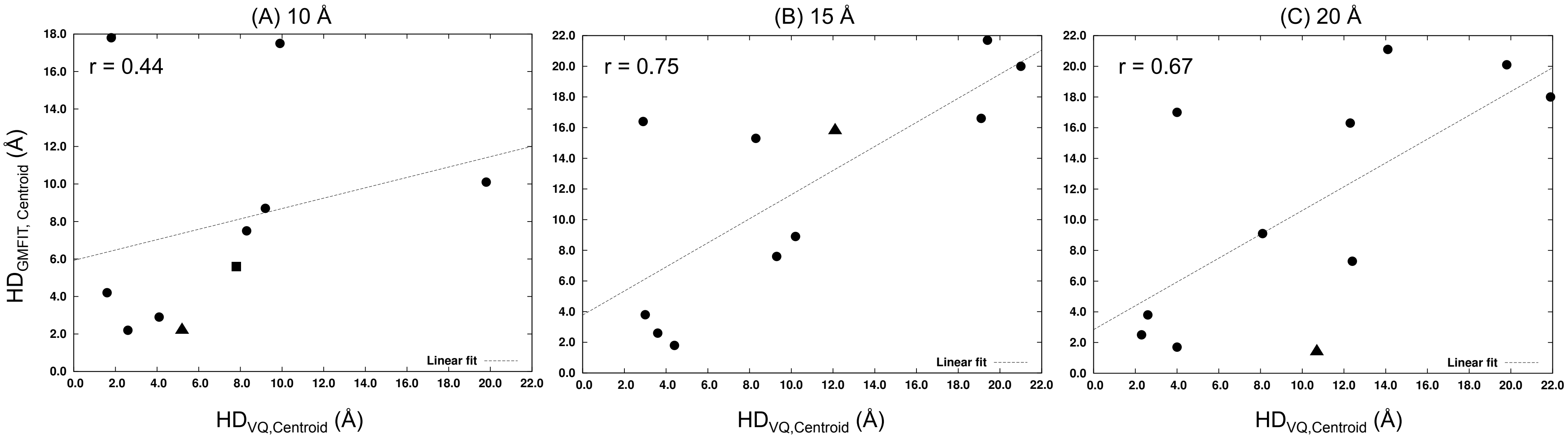

Figure S1, Related to Figure 4. Comparison of feature points obtained using vector quantisation (VQ) and GMFIT method. (A-C) The linear relationship between $HD_{VQ,Centroid}$ (Hausdorff distance calculated between the VQ points set of the density map and the point set calculated from the centroids of the native assembly components) and $HD_{GMFIT,Centroid}$ (Hausdorff distance between the GMFIT points set of the density map and the point set calculated from the centroids of the native assembly components) is shown for 10, 15 and 20 Å resolutions maps. Data points for the experimental cases 2P4N (resolution=9.0 Å, shown as filled triangle) and 4A6J (resolution=7.2 Å, shown as filled square) have been added to (A). The data points for the experimental cases 4BIJ (resolution=16.0 Å) and 1GRU (resolution=23.5 Å), both shown as filled triangles, have been added to (B) and (C), respectively. The best fitting regression line (linear fit) along with the Pearson's correlation coefficient (r) are indicated on the plots.

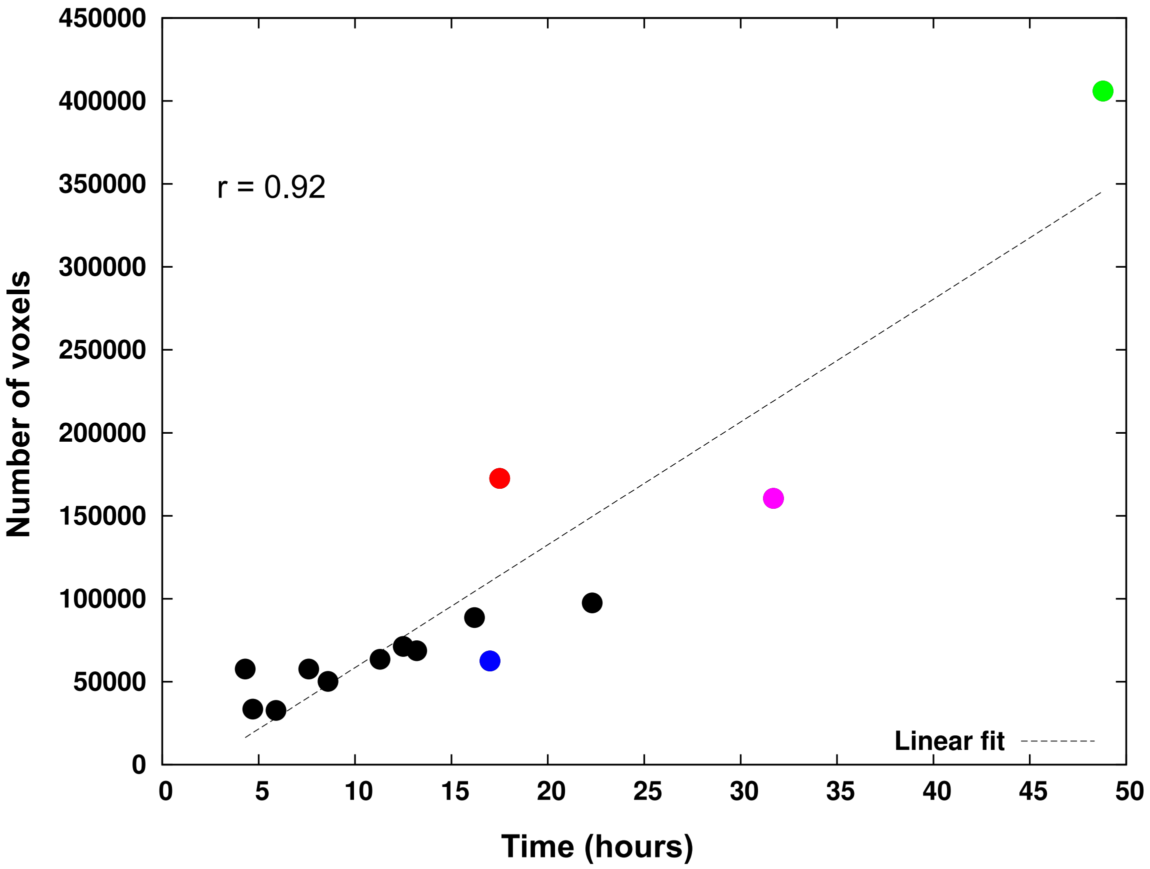

Figure S2, Related to Figure 6. Running time of g-TEMPy genetic algorithm. The number of voxels in the density map is plotted against the processing time (in hours) for the test cases in the simulated benchmark at 20 Å resolution and in the experimental benchmark. The best fitting regression line (linear fit) along with the Pearson's correlation coefficient (r = 0.92) are indicated on the plot. The data points for the experimental benchmark 2P4N, 4A6J, 4BIJ and 1GRU are shown in red, green, blue and magenta, respectively.

Table S1, Related Table 1. Component placement score for the simulated benchmark

Test case	C1	C2	C3	C4	C5	C6	C7	C8
**(A) 10Å: CPS (Å, °) for BP**								
1CS4	A: 1.6, 25.0	B: 1.8, 9.0	C: 0.8, 11.1	-	-	-	-	-
2DQJ	L: 1.1, 12.1	H: 1.7, 11.9	Y: 4.1, 22.5	-	-	-	-	-
1VCB	A: 2.1, 16.9	B: 8.1, 30.2	C: 5.5, 38.1	-	-	-	-	-
2BO9	A: 0.8, 5.7	C: 0.6, 4.8	B: 1.8, 14.5	D: 2.6, 28.1	-	-	-	-
1GPQ	A: 1.6, 14.0	B: 1.0, 13.4	C: 1.2, 20.4	D: 1.6, 14.0	-	-	-	-
2BBK	M: 6.8, 17.6	L: 8.4, 175.4	H: 3.5, 17.5	J: 3.7, 8.9	-	-	-	-
1MDA[a]	J: 2.4, 11.0	H: 6.7, 27.5	M: 19.6, 82.2	L: 4.2, 15.9	B: 8.6, 167.1	A: 4.8, 175.8	-	-
1SGF	G: 3.2, 12.3	Z: 3.3, 177.6	B: 10.7, 32.9	Y: 27.8, 81.4	X: 0.9, 11.1	A: 1.3, 36.9	-	-
1TYQ[a]	A: 3.5, 172.8	B: 3.9, 14.7	C: 0.9, 69.6	D: 0.8, 15.5	E: 6.8, 39.1	F: 9.5, 162.8	G: 6.6, 23.7	-
2GC7[a]	E: 4.5, 63.4	A: 3.4, 8.8	H: 0.7, 13.5	D: 1.0, 13.5	F: 9.1, 158.4	B: 7.0, 176.9	C: 5.8, 177.4	G: 5.6, 4.4
**(B) 10Å: CPS (Å, °) for HS**								
1CS4	A: 1.6, 25.0	B: 1.8, 9.0	C: 0.8, 11.1	-	-	-	-	-
2DQJ	L: 1.1, 14.5	H: 1.7, 16.5	4.1, 23.1	-	-	-	-	-
1VCB	A: 2.1, 29.2	B: 30.9, 85.4	C: 23.8, 159.2	-	-	-	-	-
2BO9	A: 0.8, 15.5	C: 0.6, 4.8	B: 1.8, 4.5	D: 2.1, 173.4		-	-	-
1GPQ	A: 1.6, 11.5	B: 1.0, 13.6	C: 1.1, 14.9	D: 1.8, 25.6	-	-	-	-
2BBK	M: 6.1, 178.1	L: 8.3, 162.1	H: 3.5, 12.5	J: 3.7, 5.7	-	-	-	-
1MDA[a]	J: 2.4, 11.0	H: 6.7, 27.5	M: 19.6, 82.2	L: 4.2, 15.9	B: 8.6, 167.1	A: 4.8, 175.8	-	-
1SGF	G: 3.3, 165.9	Z: 3.5, 120.4	B: 10.9, 94.8	Y: 27.9, 122.5	X: 0.7, 15.7	A: 1.5, 4.8	-	-
1TYQ[a]	A: 3.5, 65.0	B: 72.9, 145.3	C: 1.0, 11.2	D: 0.8, 11.4	E: 79.0, 91.5	F: 9.8, 133.0	G: 6.6, 157.6	-
2GC7[a]	E: 4.4, 35.2	A: 3.6, 43.9	H: 0.7, 3.8	D: 1.0, 9.7	F: 37.6, 121.6	B: 23.4, 170.4	C: 54.2, 161.7	G: 6.7, 160.9
**(C) 15Å: CPS (Å, °) for BP**								
1CS4	A: 2.2, 9.2	B: 2.9, 13.3	C: 0.6, 3.8	-	-	-	-	-
2DQJ	L: 1.1, 13.0	H: 1.6, 5.8	Y: 3.6, 11.2	-	-	-	-	-
1VCB	A: 2.9, 162.9	B: 9.2, 169.1	C: 6.6, 18.6	-	-	-	-	-
2BO9	A: 3.6, 22.0	C: 4.5, 19.5	B: 3.6, 16.2	D: 2.4, 28.3	-	-	-	-
1GPQ	A: 2.9, 43.7	B: 2.8, 26.3	C: 2.5, 26.4	D: 3.3, 53.6	-	-	-	-
2BBK	M: 10.0, 71.9	L: 9.8, 80.9	H: 4.7, 37.6	J: 3.8, 39.5	-	-	-	-
1MDA[a]	J: 2.4, 7.0	H: 3.3, 22.7	M: 8.1, 78.1	L: 5.1, 41.5	B: 7.2, 24.8	A: 7.0, 126.1	-	-
1SGF	G: 1.8, 18.7	Z: 2.2, 179.0	B: 11.7, 32.9	Y: 21.5, 38.8	X: 2.9, 11.3	A: 1.0, 173.6	-	-
1TYQ[a]	A: 47.7, 128.2	B: 45.0, 158.4	C: 1.7, 10.0	D: 4.0, 158.7	E: 5.5, 174.2	F: 46.9, 153.0	G: 46.4, 102.3	-
2GC7[a]	E: 6.2, 39.4	A: 3.0, 40.0	H: 2.3, 23.3	D: 1.9, 178.6	F: 23.3, 75.4	B: 18.0, 149.3	C: 36.4, 150.6	G: 29.8, 154.2

**(D) 15Å: CPS (Å, °) for HS**								
1CS4	A: 2.1, 9.0	B: 2.9, 17.1	C: 0.6, 3.8	-	-	-	-	-
2DQJ	L: 1.1, 13.0	H: 1.6, 5.8	Y: 3.6, 11.2	-	-	-	-	-
1VCB	A: 42.4, 54.6	B: 17.8, 149.7	C: 23.1, 162.3	-	-	-	-	-
2BO9	A: 3.7, 7.1	C: 4.4, 11.6	B: 3.6, 7.1	D: 2.7, 171.8	-	-	-	-
1GPQ	A: 3.0, 173.0	B: 2.9, 156.8	C: 2.7, 13.1	D: 2.9, 19.6	-	-	-	-
2BBK	M: 9.3, 140.9	L: 9.1, 158.8	H: 5.1, 159.5	J: 4.1, 172.0	-	-	-	-
1MDA[a]	J: 2.4, 7.0	H: 3.3, 23.9	M: 8.1, 175.7	L: 57.1, 163.2	B: 59.1, 127.3	A: 7.5, 74.2	-	-
1SGF	G: 1.7, 23.2	Z: 1.7, 22.2	B: 12.0, 158.5	Y: 21.5, 142.1	X: 3.6, 176.5	A: 0.3, 6.4	-	-
1TYQ[a]	A: 5.1, 45.5	B: 45.2, 145.7	C: 1.8, 18.9	D: 4.8, 17.5	E: 69.5, 86.2	F: 35.0, 145.8	G: 101.4, 93.8	-
2GC7[a]	E: 6.2, 39.4	A: 3.0, 40.0	H: 2.3, 23.3	D: 1.9, 178.6	F: 23.3, 75.4	B: 18.0, 149.3	C: 36.4, 150.6	G: 29.8, 154.2
**(E) 20Å: CPS (Å, °) for BP**								
1CS4	A: 4.0, 13.5	B: 3.9, 13.7	C: 1.7, 14.3	-	-	-	-	-
2DQJ	L: 1.1, 18.0	H: 1.0, 18.5	Y: 2.4, 14.1	-	-	-	-	-
1VCB	A: 4.3, 33.0	B: 33.2, 95.1	C: 21.7, 89.6	-	-	-	-	-
2BO9	A: 3.1, 24.3	C: 1.5, 26.1	B: 4.0, 33.9	D: 2.3, 23.2	-	-	-	-
1GPQ	A: 1.8, 161.3	B: 2.4, 21.5	C: 2.6, 21.3	D: 2.0, 4.7	-	-	-	-
2BBK	M: 7.5, 47.4	L: 8.0, 21.7	H: 3.7, 24.3	J: 2.8, 10.7	-	-	-	-
1MDA[a]	J: 4.4, 61.6	H: 6.0, 54.1	M: 23.1, 122.9	L: 12.0, 130.6	B: 32.5, 125.8	A: 37.3, 114.0	-	-
1SGF	G: 1.2, 40.0	Z: 2.2, 13.0	B: 12.0, 117.2	Y: 19.5, 161.0	X: 1.9, 178.2	A: 2.9, 11.8	-	-
1TYQ[a]	A: 33.0, 97.3	B: 37.8, 97.5	C: 2.1, 87.2	C: 38.9, 86.4	E: 5.5, 23.4	F: 21.4, 124.7	G: 51.0, 178.1	-
2GC7[a]	E: 4.5, 35.1	A: 6.4, 26.6	H: 2.0, 102.7	D: 2.1, 108.7	F: 12.1, 101.8	B: 8.7, 177.3	C: 9.2, 109.1	G: 10.3, 121.5
**(F) 20Å: CPS (Å, °) for HS**								
1CS4	A: 25.7, 179.6	B: 26.0, 122.4	C: 1.7, 9.2	-	-	-	-	-
2DQJ	L: 1.1, 18.0	H: 1.0, 18.5	Y: 2.4, 14.1	-	-	-	-	-
1VCB	A: 44.1, 161.3	B: 12.6, 148.9	C: 37.8, 179.2	-	-	-	-	-
2BO9	A: 3.0, 8.7	C: 1.4, 9.1	B: 3.9, 177.1	D: 2.3, 6.3	-	-	-	-
1GPQ	A: 1.8, 157.5	B: 2.3, 139.1	C: 2.5, 11.5	D: 2.2, 176.0	-	-	-	-
2BBK	M: 7.4, 11.8	L: 7.2, 164.9	H: 3.7, 20.6	J: 2.8, 27.0	-	-	-	-
1MDA[a]	J: 4.4, 60.0	H: 5.9, 56.2	M: 26.3, 121.3	L: 12.0, 176.8	B: 8.2, 158.6	A: 63.1, 174.9	-	-
1SGF	G: 1.1, 12.9	Z: 2.2, 20.3	B: 20.4, 118.5	Y: 12.5, 176.0	X: 1.9, 161.9	A: 3.1, 62.9	-	-
1TYQ[a]	A: 32.8, 178.8	B: 38.7, 111.2	C: 2.3, 112.1	D: 39.4, 153.0	E: 96.0, 160.6	F: 74.5, 110.9	G: 51.0, 168.1	-
2GC7[a]	E: 4.9, 166.1	A: 6.9, 177.2	H: 28.1, 170.0	D: 2.2, 31.6	F: 11.5, 179.4	B: 8.7, 170.5	C: 9.6, 79.6	G: 27.4, 133.2

Description of the items: Test case, the PDB ID of the assemblies; BP, the best predicted assembly with the lowest average Cα RMSD from the native among 20 GA runs; HS, the highest scoring assembly among 20 GA runs; CPS, the component placement score describing the shift in Å and rotation in degrees needed to superpose the individual predicted components onto their corresponding native components; C1-8 describes the CPS score for the individual components in the assembly along with their chain ID's; [a]N-terminal residues have been removed in: 1MDA chain H and J (1-31), 2GC7 chain A and E (5-44) and 1TYQ chain G (11-27).

Table S2, Related to Table 2. Component placement score for the experimental benchmark

(A) CPS (Å, °) for BP							
**Fitted PDB ID**	**C1**	**C2**	**C3**	**C4**	**C5**	**C6**	**C7**
2P4N	K: 3.2, 19.3	A: 5.3, 21.6	B: 4.6, 13.9	-	-	-	-
4A6J	E: 7.9, 22.3	F: 4.0, 12.4	G: 1.1, 12.2	H: 5.1, 11.7	-	-	-
4BIJ	A: 12.1, 22.2	B: 11.7, 22.6	C: 11.3, 15.2	D: 10.0, 21.7	E: 11.2, 17.6	-	-
1GRU	H: 8.0, 12.6	I: 9.7, 26.8	J: 7.7, 20.0	K: 9.2, 26.0	L: 8.2, 6.7	M: 10.7, 17.0	N: 9.2, 37.7
**(B) CPS (Å, °) for HS**							
2P4N	K: 3.2, 14.4	A: 5.2, 153.1	B: 4.5, 13.3	-	-	-	-
4A6J	E: 7.9, 22.3	F: 4.0, 12.4	G: 1.1, 12.2	H: 5.1, 11.7	-	-	-
4BIJ	A: 12.1, 22.2	B: 11.7, 22.6	C: 11.3, 15.2	D: 10.0, 21.7	E: 11.2, 17.6	-	-
1GRU	H: 8.0, 25.7	I: 9.7, 38.3	J: 7.7, 12.6	K: 9.3, 31.7	L: 8.3, 28.0	M: 10.7, 35.2	N: 9.1, 23.9

Description of the items: Test case, the PDB ID of the assemblies; BP, the best predicted assembly with the lowest average Cα RMSD from the native among 20 GA runs; HS, the highest scoring assembly among 20 GA runs; CPS, the component placement score describing the shift in Å and rotation in degrees needed to superpose the individual predicted components onto their corresponding native components; Component1-8 describes the CPS score for the individual components in the assembly along with their chain ID's.

Table S3, Related to Table1. Summary of model accuracy for the 10 Å simulated maps using the centroids of the native assembly components as the starting positions

(A) Model accuracy scores								
NC	Test case	BP			HS			Rank of BP
		TS	APS (Å, °)	RMSD (Å)	TS	APS (Å, °)	RMSD (Å)	
3	1CS4	1.0	0.1, 8.9	2.4	1.0	0.1, 8.9	2.4	1
	2DQJ	1.0	0.1, 12.5	2.3	1.0	0.1, 12.5	2.3	1
	1VCB	1.0	0.1, 14.7	3.0	1.0	0.1, 21.3	4.1	2
4	2BO9	1.0	0.1, 9.1	2.3	1.0	0.1, 9.1	2.3	1
	1GPQ	1.0	0.1, 11.2	2.2	1.0	0.1, 11.2	2.2	1
	2BBK	1.0	0.1, 7.7	1.8	1.0	0.1, 7.7	1.8	1
6	1MDA[a]	1.0	0.1, 13.2	2.5	1.0	0.1, 13.2	2.5	1
	1SGF	1.0	0.2, 9.4	4.5	1.0	0.2, 9.4	4.5	1
7	1TYQ[a]	1.0	0.4, 49.6	9.1	1.0	0.3, 44.8	10.8	8
8	2GC7[a]	1.0	0.1, 17.6	3.6	1.0	0.4, 74.3	11.0	2

(B) CPS for BP (Å, °)								
Test case	C1	C2	C3	C4	C5	C6	C7	C8
1CS4	A: 0.1, 6.6	B: 0.0, 9.0	C: 0.0, 11.1	-	-	-	-	-
2DQJ	L: 0.1, 14.4	H: 0.2, 16.5	Y: 0.1, 6.6	-	-	-	-	-
1VCB	A: 0.1, 23.8	B: 0.1, 12.8	C: 0.0, 7.6	-	-	-	-	-
2BO9	A: 0.1, 16.3	C: 0.0, 4.8	B: 0.1, 10.3	D: 0.1, 5.0	-	-	-	-
1GPQ	A: 0.1, 9.3	B: 0.0, 13.3	C: 0.1, 4.7	D: 0.2, 17.6	-	-	-	-
2BBK	M: 0.2, 15.0	H: 0.1, 5.3	H: 0.0, 5.6	J: 0.0, 4.8	-	-	-	-
1MDA[a]	J: 0.1, 5.5	H: 0.1, 6.5	M: 0.2, 34.1	L: 0.2, 14.3	B: 0.1, 7.3	A: 0.1, 11.4	-	-
1SGF	G: 0.2, 11.7	Z: 0.1, 12.7	B: 0.2, 8.2	Y: 0.2, 6.9	X: 0.3, 11.6	A: 0.1, 5.1	-	-
1TYQ[a]	A: 0.0, 22.1	B: 1.1, 168.8	C: 0.2, 20.1	D: 0.2, 25.6	E: 0.1, 16.0	F: 0.7, 71.0	G: 0.1, 23.6	-
2GC7[a]	E: 0.1, 20.3	A: 0.1, 15.4	H: 0.2, 21.0	D: 0.1, 22.2	F: 0.1, 13.7	B: 0.1, 29.4	C: 0.1, 6.2	G: 0.2, 13.0
(C) CPS for HS (Å, °)								
1CS4	A: 0.1, 6.6	0.0, 9.0	0.0, 11.1	-	-	-	-	-
2DQJ	L: 0.1, 14.4	H: 0.2, 16.5	Y: 0.1, 6.6	-	-	-	-	-
1VCB	A: 0.1, 29.0	B: 0.1, 15.8	C: 0.1, 19.1	-	-	-	-	-
2BO9	A: 0.1, 16.3	C: 0.0, 4.8	B: 0.1, 10.3	D: 0.1, 5.0	-	-	-	-
1GPQ	A: 0.1, 9.3	B: 0.0, 13.3	C: 0.1, 4.7	D: 0.2, 17.6	-	-	-	-
2BBK	M: 0.2, 15.0	H: 0.1, 5.3	H: 0.0, 5.6	J: 0.0, 4.8	-	-	-	-

1MDA[a]	J: 0.1, 5.5	H: 0.1, 6.5	M: 0.2, 34.1	L: 0.2, 14.3	B: 0.1, 7.3	A: 0.1, 11.4	-	-
1SGF	G: 0.2, 11.7	Z: 0.1, 12.7	B: 0.2, 8.2	Y: 0.2, 6.9	X: 0.3, 11.6	A: 0.1, 5.1	-	-
1TYQ[a]	A: 0.1, 18.0	B: 0.1, 17.2	C: 0.5, 54.0	D: 0.3, 27.1	E: 0.1, 12.4	F: 1.1, 164.2	G: 0.1, 20.9	-
2GC7[a]	E: 0.1, 11.5	A: 0.5, 173.0	H: 0.2, 13.5	D: 0.1, 11.7	F: 0.1, 7.5	B: 0.2, 26.7	C: 0.9, 175.0	G: 1.1, 175.9

Description of the items: NC, the number of components in the assembly; Test case, the PDB ID of the assemblies; BP, the best predicted assembly with the lowest average Cα RMSD from the native among 20 GA runs; HS, the highest scoring assembly among 20 GA runs; TS, the topology score describing the fraction of components placed correctly; APS, the assembly placement score describing the average shift in Å and rotation in degrees needed to superpose all the predicted components onto their corresponding native components; RMSD, the average Cα RMSD between the predicted components and its corresponding native components; CPS, the component placement score describing the shift in Å and rotation in degrees needed to superpose the individual predicted components onto their corresponding native components; Rank of BP, the rank of the BP among 20 GA predictions based on the fitness function value. Component1-8 describes the CPS score for the individual components in the assembly along with their chain ID's; [a]N-terminal residues have been removed in: 1MDA chain H and J (1-31), 2GC7 chain A and E (5-44) and 1TYQ chain G (11-27).

Table S4, Related to Table 1. Summary of model accuracy for the 20 Å simulated maps using the centroids of the native assembly components as the starting positions

(A) Model accuracy scores								
NC	Test case	BP			HS			Rank of BP
		TS	APS (Å, °)	RMSD (Å)	TS	APS (Å, °)	RMSD (Å)	
3	1CS4	1.0	0.1, 16.1	4.0	1.0	0.1, 16.1	4.0	1
	2DQJ	1.0	0.1, 12.5	2.2	1.0	0.1, 12.5	2.2	1
	1VCB	1.0	0.1, 14.2	2.6	1.0	0.1, 14.2	2.6	1
4	2BO9	1.0	0.1, 13.3	3.2	1.0	0.1, 13.3	3.2	1
	1GPQ	1.0	0.1, 13.4	2.6	1.0	0.2, 46.5	6.1	10
	2BBK	1.0	0.1, 7.0	1.7	1.0	0.1, 7.0	1.7	1
6	1MDA[a]	1.0	0.2, 19.7	3.8	1.0	0.3, 47.3	6.7	4
	1SGF	1.0	0.2, 16.4	5.2	1.0	0.2, 16.4	5.2	1
7	1TYQ[a]	1.0	0.4, 58.2	10.8	1.0	0.4, 58.2	10.8	1
8	2GC7[a]	1.0	0.4, 77.4	11.4	0.75	21.2, 69.9	26.7	3

(B) CPS for BP (Å, °)								
Test case	C1	C2	C3	C4	C5	C6	C7	C8
1CS4	A: 0.1, 14.6	B: 0.1, 6.6	C: 0.1, 27.1	-	-	-	-	-
2DQJ	L: 0.2, 14.0	H: 0.1, 13.9	Y: 0.1, 9.7	-	-	-	-	-
1VCB	A: 0.1, 13.5	B: 0.1, 8.8	C: 0.1, 20.2	-	-	-	-	-
2BO9	A: 0.1, 21.7	C: 0.1, 13.7	B: 0.0, 13.9	D: 0.0, 3.6	-	-	-	-
1GPQ	A: 0.1, 11.4	B: 0.1, 21.3	C: 0.0, 7.6	D: 0.0, 13.4	-	-	-	-
2BBK	M: 0.2, 8.4	L: 0.1, 5.7	H: 0.1, 5.5	J: 0.1, 8.5	-	-	-	-
1MDA[a]	J: 0.2, 17.9	H: 0.1, 11.1	M: 0.2, 26.6	L: 0.2, 17.7	B: 0.2, 21.1	A: 0.2, 23.6	-	-
1SGF	G: 0.2, 10.3	Z: 0.2, 18.0	B: 0.2, 15.0	Y: 0.2, 9.7	X: 0.2, 19.1	A: 0.3, 26.3	-	-
1TYQ[a]	A: 0.1, 10.9	B: 1.0, 173.6	C: 0.9, 105.3	D: 0.2, 19.3	E: 0.3, 32.1	F: 0.5, 52.0	G: 0.1, 14.5	-
2GC7[a]	E: 0.2, 29.5	A: 0.1, 20.9	H: 0.1, 15.0	D: 0.0, 21.7	F: 0.1, 167.7	B: 0.1, 29.3	C: 0.8, 170.0	G: 0.9, 164.8
(C) CPS for HS (Å, °)								
1CS4	A: 0.1, 14.6	B: 0.1, 6.6	C: 0.1, 27.1	-	-	-	-	-
2DQJ	L: 0.2, 14.0	H: 0.1, 13.9	Y: 0.1, 9.7	-	-	-	-	-
1VCB	A: 0.1, 13.5	B: 0.1, 8.8	C: 0.1, 20.2	-	-	-	-	-
2BO9	A: 0.1, 21.7	C: 0.1, 13.7	B: 0.0, 13.9	D: 0.0, 3.6	-	-	-	-
1GPQ	A: 0.1, 11.4	B: 0.1, 17.8	C: 0.5, 150.4	D: 0.1, 6.5	-	-	-	-
2BBK	M: 0.2, 8.4	L: 0.1, 5.7	H: 0.1, 5.5	J: 0.1, 8.5	-	-	-	-

1MDA[a]	J: 0.2, 15.6	H: 0.1, 5.5	M: 0.2, 17.9	L: 0.2, 54.0	B: 0.1, 15.3	A: 1.0, 175.7	-	-
1SGF	G: 0.2, 10.3	Z: 0.2, 18.0	B: 0.2, 15.0	Y: 0.2, 9.7	X: 0.2, 19.1	A: 0.3, 26.3	-	-
1TYQ[a]	A: 0.1, 10.9	B: 1.0, 173.6	C: 0.9, 105.3	D: 0.2, 19.3	E: 0.3, 32.1	F: 0.5, 52.0	G: 0.1, 14.5	-
2GC7[a]	E: 0.1, 22.9	A: 0.1, 12.8	H: 84.3, 178.7	D: 0.0, 15.6	F: 0.1, 20.7	B: 83.8, 141.5	C: 0.9, 151.4	G: 0.1, 15.4

Description of the items: NC, the number of components in the assembly; Test case, the PDB ID of the assemblies; BP, the best predicted assembly with the lowest average Cα RMSD from the native among 20 GA runs; HS, the highest scoring assembly among 20 GA runs; TS, the topology score describing the fraction of components placed correctly; APS, the assembly placement score describing the average shift in Å and rotation in degrees needed to superpose all the predicted components onto their corresponding native components; RMSD, the average Cα RMSD between the predicted components and its corresponding native components; CPS, the component placement score describing the shift in Å and rotation in degrees needed to superpose the individual predicted components onto their corresponding native components; Rank of BP, the rank of the BP among 20 GA predictions based on the fitness function value. Component1-8 describes the CPS score for the individual components in the assembly along with their chain ID's; [a]N-terminal residues have been removed in: 1MDA chain H and J (1-31), 2GC7 chain A and E (5-44) and 1TYQ chain G (11-27).
